# Was the M_w_ 7.5 1952 Kern County, California, earthquake induced (or triggered)?

**DOI:** 10.1007/s10950-017-9685-x

**Published:** 2017-10-02

**Authors:** Susan E. Hough, Victor C. Tsai, Robert Walker, Fred Aminzadeh

**Affiliations:** 1 U.S. Geological Survey, Pasadena, CA USA; 2 California Institute of Technology, Pasadena, CA USA; 3 University of Southern California, Los Angeles, CA USA

**Keywords:** Induced earthquakes, Triggered earthquakes, Kern County earthquake

## Abstract

Several recent studies have presented evidence that significant induced earthquakes occurred in a number of oil-producing regions during the early and mid-twentieth century related to either production or wastewater injection. We consider whether the 21 July 1952 M_w_ 7.5 Kern County earthquake might have been induced by production in the Wheeler Ridge oil field. The mainshock, which was not preceded by any significant foreshocks, occurred 98 days after the initial production of oil in Eocene strata at depths reaching 3 km, within ~1 km of the White Wolf fault (WWF). Based on this spatial and temporal proximity, we explore a potential causal relationship between the earthquake and oil production. While production would have normally be expected to have reduced pore pressure, inhibiting failure on the WWF, we present an analytical model based on industry stratigraphic data and best estimates of parameters whereby an impermeable splay fault adjacent to the main WWF could plausibly have blocked direct pore pressure effects, allowing the poroelastic stress change associated with production to destabilize the WWF, promoting initial failure. This proof-of-concept model can also account for the 98-day delay between the onset of production and the earthquake. While the earthquake clearly released stored tectonic stress, any initial perturbation on or near a major fault system can trigger a larger rupture. Our proposed mechanism provides an explanation for why significant earthquakes are not commonly induced by production in proximity to major faults.

**Electronic supplementary material:**

The online version of this article (doi:10.1007/s10950-017-9685-x) contains supplementary material, which is available to authorized users.

## Introduction

The hazard associated with induced earthquakes has come to the fore in recent years with the rise in seismic activity, including events with magnitudes upwards of 4, in areas where there has been an increase in subsurface fluid injection and production operations, including both hydraulic fracturing (Schulz et al. 2015; Atkinson et al. 2016) and injection of large volumes of wastewater (Keranen et al. 2013; Goebel et al. 2015; Walsh and Zoback 2015; Weingarten et al. 2015). While the association between earthquakes and wastewater injection was established in the late 1960s (Evans 1966), and a small number of published studies in the twentieth century identified potentially induced events associated with fossil fuel production (Caloi et al. 1956; Kovach 1974; Simpson and Leith 1985; Nicholson and Wesson 1990), the level of hazard associated with induced earthquakes has only in recent years been evaluated (Petersen et al. 2016). Several recent studies have presented evidence suggesting that oil and gas production and/or wastewater injection may have caused damaging induced events as early as the early- to mid-twentieth century (Hough and Page 2015; Frohlich et al. 2016; Hough and Page 2016). In this paper, we explore the possibility that the 1952 Kern County, California, earthquake, estimated magnitude 7.3–7.7, might have been induced by oil production in the Wheeler Ridge oil field. We first review the history of oil production in this field; we then discuss the earthquake and its possible association with production.

## The Wheeler Ridge oil field

**Fig. 1:**
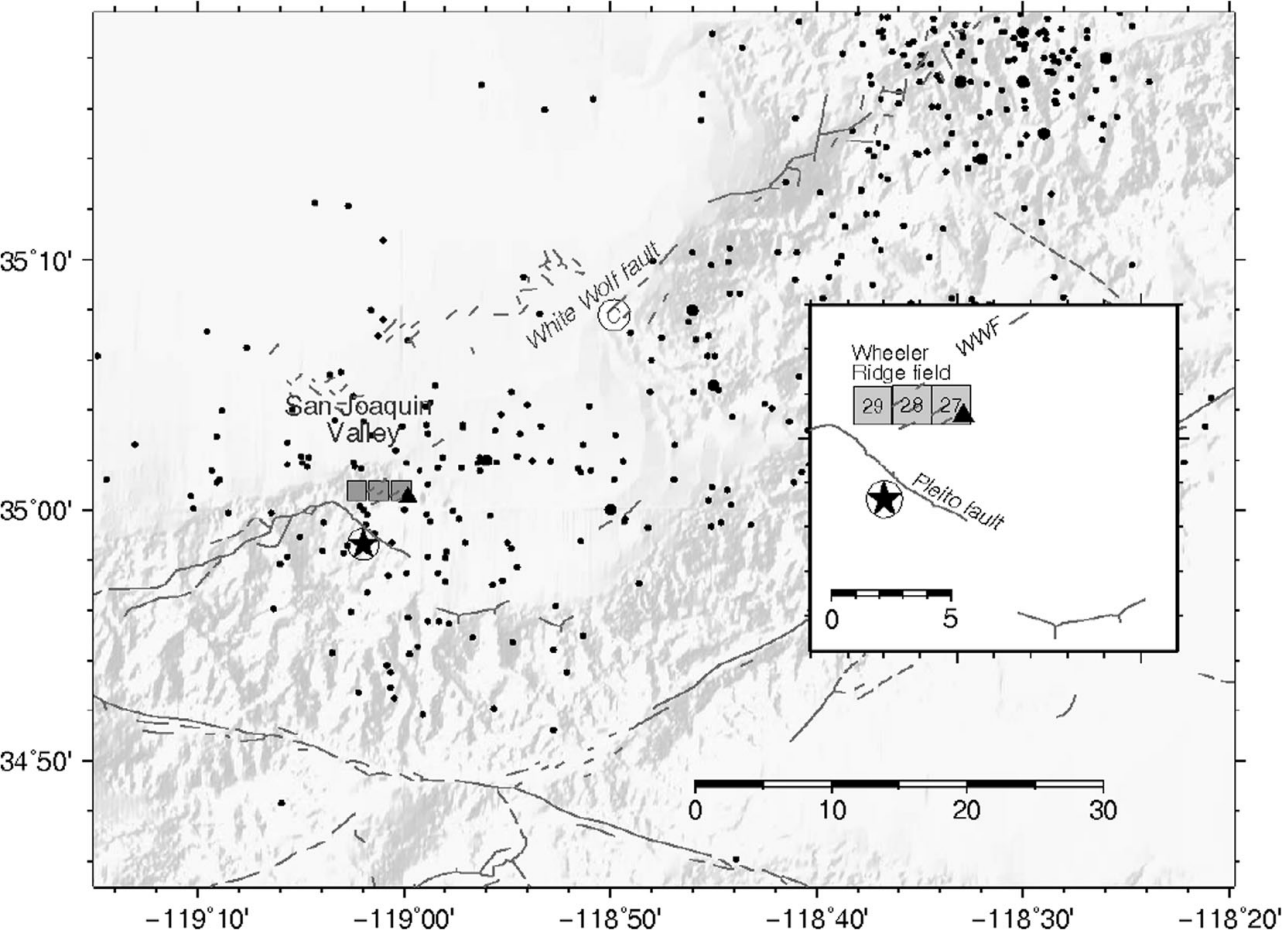
Map showing topography (90-m resolution Shuttle Radar Topographic Mission digital elevation model), locations of Wheeler Ridge oil field operations (*small gray squares*) in the early 1950s, preferred epicenter of the 1952 Kern County earthquake (*black star*; see electronic supplement), aftershock locations from the Southern California Seismic Network (SCSN) catalog (*small dots*) and Dreger and Savage (1999) (*larger black dots*), location of Comanche Point (*circle labeled C*), and location of a 1939 M_w_4.6 earthquake (*black triangle*; Ishida and Kanamori 1980). *Inset* shows sections within the Wheeler Ridge oil field, together with the preferred epicenters of the 1952 and 23 Feb. 1939 earthquakes (*same symbols* as in main figure)

**Fig. 2:**
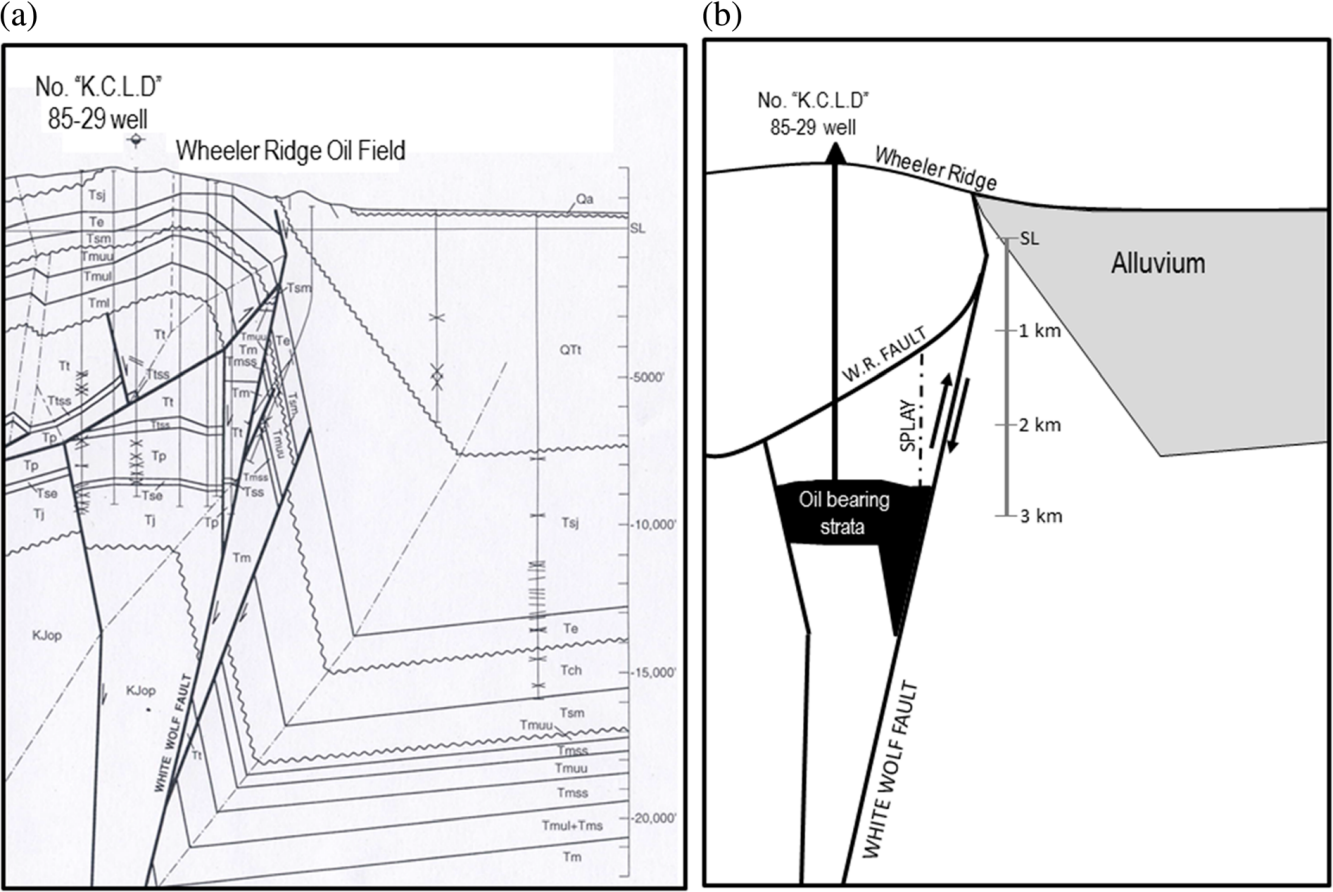
(**a**, l*eft*) Cross section (south to left, north to right; no vertical exaggeration) of the Wheeler Ridge oil field (*left*) (Davis et al. 1996). Well "K.C.L.D" 85-29 struck oil in the Eocene sandstone unit labeled Tj (and indicated in **b**). Precise well locations and lithologic contacts are constrained from industry records; wellhead locations were surveyed to an accuracy within 1 m (see electronic supplement). Regional faults (light lines) from Jennings (1994). **b** Schematic representation of cross section indicating Eocene production layer within Tj unit (*right*), including the Wheeler Ridge fault (WR fault) and White Wolf fault. *Dashed line* indicates splay fault identified in cross section

Wheeler Ridge is a prominent topographic feature near the southern end of the San Joaquin valley in central California (Figs. 1 and 2), the surface expression of an anticline that had first been a target for oil exploration in the early 1920s (Collum 1923). Before 1952, producing horizons within the Wheeler Ridge oil field were limited to shallow (depths <305 m) Miocene sandstones within the two square sections labeled 27 and 28 in Fig. 1 (Carls 1951; Walling 1952). Under the Public Land Survey system used to subdivide land in most of the USA, a section is nominally 2.6 km^2^ (1 mile^2^) within a grid of Townships and Ranges. The sections of interest for this study are within Township 11 N, Range 20 W. Cross sections generated from surface and subsurface data revealed the asymmetry of the Wheeler Ridge anticline, leading to deep drilling along the south limb of the anticline in the late 1930s (Musser 1939). A test well in section 28 (see electronic supplement) reached a reported depth of 2656 m (8713 ft) in March, 1938 (reported non-SI units indicated) (Musser 1939). This well was later deepened to 3404 m (11,168 ft), then plugged back to 2561 m (8402 ft) in September, 1939 (Musser 1940), although the State report does not indicate when the well was deepened. Deeper exploration led to the discovery of the Wheeler Ridge thrust fault, the footwall of which was explored for oil prospects soon thereafter (Davis et al. 1996). Drilling in the Coal Oil Canyon, in the northeast corner of section 29 (Fig. 1), began in the late 1940s, striking oil at approximately 305 m (1000′) in May, 1948. Deeper drilling ensued, with unexpected shallow strikes in the Coal Oil Canyon region in 1951. By the end of 1951, cumulative oil production within the field had reached 5,593,715 barrels (bbl; see electronic supplement), with annual production of approximately 270,000 bbl (COF 1951a, b). According to the then Deputy Supervisor of the State Division of Oil and Gas, the most notable discovery during 1952 within the San Joaquin Valley, which by that time included the Wheeler Ridge region as well as larger fields, was made by Richfield Oil Corporation with the completion of a well (No. “K.C.L.D” 85-29) located at 35.01198N, 119.03033W in the northeast corner of section 29 (Fig. 1; electronic supplement). Initial production was 1170 bbl/day, from the Metralla Sandstone Member of the Eocene Tejon Formation at a reported 2926 to 2974 m (9600 to 9756 ft) depth (Walling 1952). This well was completed on 14 April 1952 and immediately placed on production, only to immediately show signs of abnormal annular gas pressure that required remedial work starting 20 April 1952. Normal production commenced on 14 May 1952, although on June 11, 1952, it was reported in the media that production was “being restricted to 400 barrels daily but with difficulty because of enormous pressures in the well which continue to creep upward” (Sullivan 1952). Detailed industry data on well-head production rates and pressures have not been found. By the end of 1952, six additional wells had been completed or were underway in section 29, including one completed prior to 21 July 1952.

The Eocene production layer lies beneath the blind Wheeler Ridge thrust fault (Fig. 2b; Davis et al. 1996), a south-dipping thrust fault associated with a complex series of folds and scarps (Keller et al. 1989). The south-west-dipping Pleito fault (Fig. 1) may connect at depth with the Wheeler Ridge fault (Keller et al. 1989). Prior to 1952, production in the Wheeler Ridge oil field had been limited to formations above the Wheeler Ridge fault. Production within the Wheeler Ridge oil field comes from the Eocene-age Metralla Sandstone Member of the Tejon (Tj) Formation, which comprises a sequence of interbedded, generally fine- to medium-grained sandstones and siltstones (Carls 1951). The Tj formation directly abuts strands of the White Wolf fault (WWF) at depths of 2–4 km. The thickness of productive reservoir sandstones ranges from 6 to 60 m (Davis et al. 1996); at the K.C.L.D. well 85-29, a thickness of 47 m (154 ft) is constrained from drilling logs.

## The 21 July 1952 Kern County, California, earthquake

The 1952 Kern County earthquake is the second-largest earthquake in California in the twentieth century; it was preceded by other events in the region, including a M_w_ 4.6 earthquake on 23 Feb. 1939 (Fig. 1). The 1952 earthquake has been associated with the WWF based on observed surface rupture (Buwalda and St. Amand 1955), geodetic data (Bawden 2001), and seismic data (Dreger and Savage 1999; Ishida and Kanamori 1980; Gutenberg 1955a; Richter 1955; Båth and Richter 1958). The mainshock is relatively well characterized, including an instrumentally determined epicenter estimated from the sparse regional network and mapped surface rupture indicating unilateral propagation to the northeast of this epicenter (Buwalda and St. Armand 1955). The precise magnitude, epicenter, and hypocentral depth, however, remain uncertain due to data limitations. Ben-Menahem (1978) estimated Mw 7.3 based on analysis of teleseismic surface waves. Gutenberg (1955b) estimated M_L_7.7. The current US Geological Survey catalog estimate is Mw7.5. Our preferred epicenter is 34.977N, 119.033W (Gutenberg 1955a; Ishida and Kanamori 1980; Felzer 2013; Hutton et al. 2010; Hutton, pers. comm. 2016; see electronic supplement for details ).

Mainshock hypocentral depth is effectively unconstrained by available data; Ishida and Kanamori (1980) simply state that it cannot be determined (see electronic supplement). Bawden (2001) used available geodetic data to investigate fault geometry and slip distribution; his preferred model includes a two-segment right-stepping rupture along the steeply dipping (dip 75°) WWF. Left-lateral oblique rupture on the westernmost, epicentral segment is inferred to be deeper than rupture to the east, reaching estimated depths of 6–27 km (Bawden 2001). In the epicentral region, Bawden (2001) infers 3.6 m of left-lateral strike slip and 1.6 m of reverse slip. While published models agree that most of the slip on the western segment was deeper than 5 km (Bawden 2001), resolution of shallow slip is fundamentally limited by data availability; one moreover cannot assume that the nucleation point of any earthquake is close to the region of maximum slip. Furthermore, following the earthquake, three surface breaks were mapped crossing the crest of Wheeler Ridge, with a trend roughly parallel to the strike of the WWF, “cross[ing] hills and depressions indifferently.” (Buwalda and St. Amand 1955). The description of these features, with a maximum displacement of 1.3 m, suggests them to have been tectonic in origin, which in turn suggests that significant shallow moment release occurred on the inferred epicentral segment of the rupture (see electronic supplement).

Johnston (1955; page 221) presents a brief summary of damage to oil fields caused by the Kern County mainshock, stating only that “relatively little effect of the shock could be detected in any of the wells” within the Wheeler Ridge oil field. While no details are provided (or found in other sources), “relatively little” suggests that wells in the field were disturbed to some extent. Johnston (1955) further notes that slight ground slumping around well installations caused “a great deal of pump trouble.”

## An induced (or triggered) event?

The left-lateral WWF had been identified prior to the earthquake as having likely experienced large-scale displacement (Hoots 1930), its long-term slip-rate is estimated at 2 mm/year (WGCEP 2008) with an estimated recurrence interval of 1000 years for Mw 7.3 events (Hearn et al. 2013). Prior to the 1952 earthquake, the western terminus of the fault was thought to be near Comanche Point (Fig. 1), a prominent anticlinal fold ≈20 km NE of Wheeler Ridge (Fig. 1; Hoots 1930). The occurrence of a tectonic Mw 7.5 event on this fault is thus certainly plausible, and an earthquake this large clearly released significant tectonic stress. Thus, we cannot rule out the possibility that the 1952 event was entirely tectonic in origin. The long-term slip rate is, however, lower than that of the nearby Garlock and San Andreas faults (7 and 34 mm/year, respectively; Hearn et al. 2013). It is impossible to know how soon a large earthquake might have happened on the fault, in the absence of anthropogenic triggering forces; a low slip-rate fault can remain close to failure for long periods of time (e.g., Hough et al. 2003). Before providing evidence for our hypothesis of triggering by oil production, we note that there is no evidence that the earthquake was triggered by other outside forces that have been shown to trigger earthquakes in some cases. As is typical for the region, there were only trace amounts of rain during the 3 months prior to the mainshock (see Data and Resources). The mainshock was also not preceded by any large global events or significant regional seismicity that might plausibly have triggered an earthquake by either dynamic or static stress change (see Data and Resources). In the month prior to the earthquake, no earthquake larger than estimated Mw 6.8 was recorded, and recorded earthquake activity in southern California was unremarkable.

Available data do, however, establish a temporal and spatial association between the nucleation point of the 1952 rupture and initial production from a deep Eocene formation within the Wheeler Ridge oil field. While the mainshock rupture clearly released stored tectonic stress, given the low slip rates and long recurrence intervals indicated for the WWF, the close spatial and temporal association suggests that the timing and location of the 1952 event were controlled at least in part by hydrocarbon production. The 1952 mainshock occurred 98 days after the initiation of production from a well drilled into Eocene strata at depths (below ground surface) of approximately 3 km, at a distance of ≈1 km from the WWF. The preferred mainshock epicenter is within ≈5 km of this well, and inferred surface rupture is within ≈2 km of the well. The 23 Feb. 1939 M4.6 earthquake (Fig. 1) also occurred in proximity to the Wheeler Ridge oil field following a deepening of wells that began in 1938 and continued through 1939 (Musser 1939). We further note that, prior to 1952, production was concentrated within sections 27 and 28 (see Fig. 1), while the exploration and production that began in 1952 were in section 29, closer to the WWF. Given both the infrequency of large tectonic events on the WWF and the temporal and spatial proximity of the 1952 earthquake to production, we explore whether a physical model constrained by industry data further supports a causal relationship between the earthquake and industry activities.

**Fig. 3:**
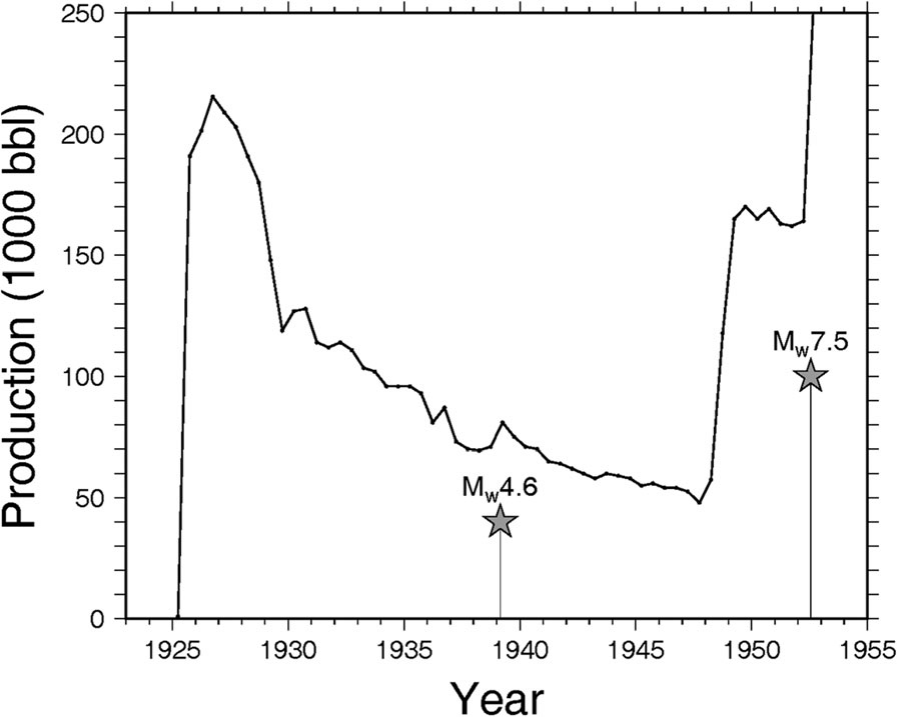
Biannual fluid (oil and water) production in the Wheeler Ridge oil field, in 1000s of barrels (i.e., 1000s of barrels per 6 months). Dates and magnitudes of the 1952 Kern County earthquake and a M_w_4.6 event in 1939 are indicated (*stars*). Early production was from shallow depths (<1000′), within sections 27 and 28 (see Fig. 1); from 1948 onwards, production was concentrated in section 29

Several mechanisms have been proposed to explain earthquakes induced by hydrocarbon production, including isostatic adjustment following mass withdrawal (McGarr 1991). Total production in the Wheeler Ridge oil field prior to 30 June 1952 was 5.59 × 10^6^ barrels (COF 1951a), or approximately 7.5 × 10^8^ kg, assuming a density estimated from reported oil properties of 850 kg/m^3^ (Fig. 3). By comparison, total mass extraction, which was suggested to have possibly triggered the 1987 Mw5.9 Whittier earthquake in the Los Angeles Basin, was ≈1.4 × 10^11^ kg in the Montebello field for the years 1924–1987 (McGarr 1991). While it is possible that total mass extraction from shallow (<1000′) depth contributed to the occurrence of the 1952 Kern County earthquake, we ignore the influence of overall early production in the field due to the small total withdrawal (e.g., relative to the Montebello field) and shallow depth of initial production in the field, focusing instead on the possible effects of production beginning in 1952. We note that the M_w_4.6 event on 23 Feb. 1939 also occurred shortly after initial exploratory drilling of deeper strata (Musser 1939); the estimated epicenter of this event (Ishida and Kanamori 1980) is also within a few km of the deep test well in section 28 (Fig. 1).

Fluid withdrawal is expected to reduce pore pressure on nearby faults, inhibiting failure assuming a standard Coulomb-Mohr failure criterion (Segall 1989). The association between production and induced earthquakes is, however, well-established (Segall 1989; Baranova et al. 1999). In the present case, the poroelastic effects of fluid withdrawal would have involved reduced pore pressure in the formation, which would have influenced both the pore pressure on the fault and the normal stress acting on it. To explore this possible mechanism, we consider analytical solutions for the expected normal stress change associated with fluid withdrawal from a layer with thickness *H* (47 m), at time-variable distance *L(t)* from the fault (Soltanzadeh and Hawkes 2008; electronic supplement). Our analytical approach takes into account both direct pore pressure effects and geomechanical effects (i.e., expected stress change) due to production. The distance between the well and the fault, *L*_*0*_, is estimated to be 1 km. We use these solutions to consider the competing effects of pore pressure change and normal stress change. We estimate key parameters from available reports and other sources (Archie 1942; Walling 1952; Calhoun 1976; Glaso 1980; Ahmed 2006; Fitts 2013; see electronic supplement).

**Fig. 4:**
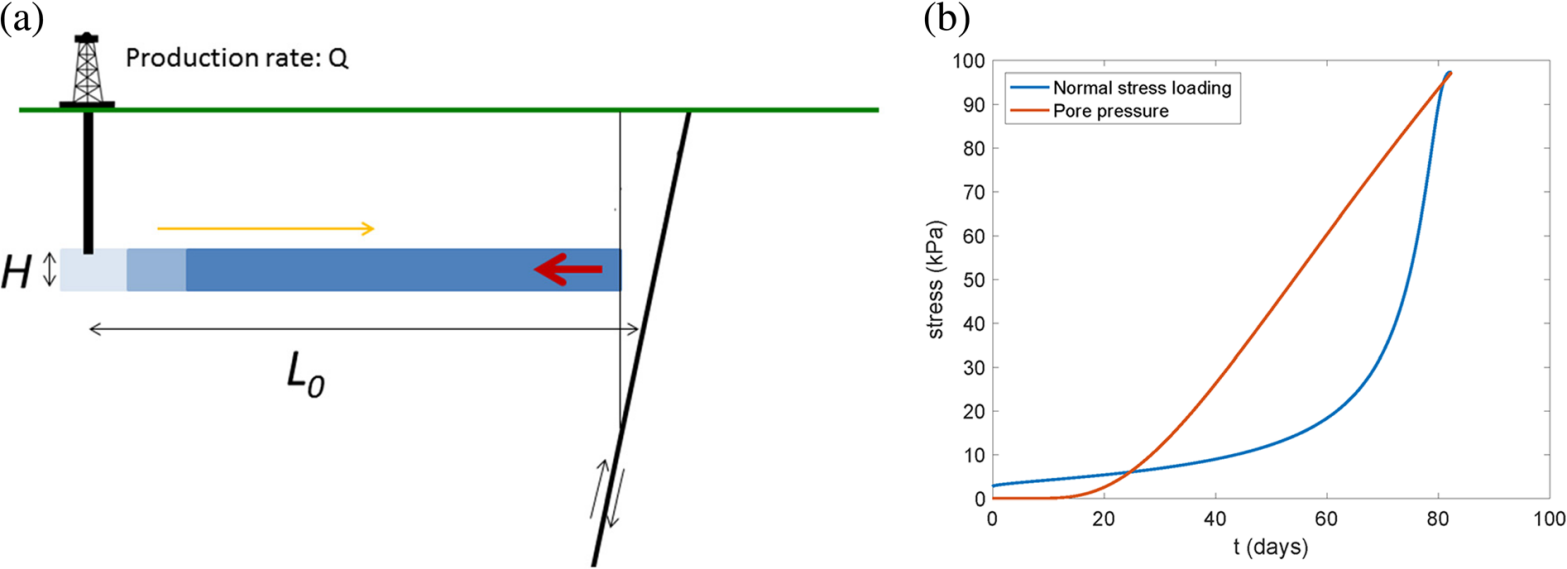
**a** Cartoon illustrating model (*left*). As a reduction in pore pressure due to oil production migrates (*yellow arrow*; L(t)) towards the WWF fault (*dark black line*), normal stress and pore pressure on the fault, are reduced (**b**, *right*). Negative of predicted normal stress change (*blue line*, indicating unclamping) and predicted pore pressure change (*orange line*) as a function of time (days) following the beginning of production from the Eocene formation, assuming *D* = 0.04 m^2^/s

As shown in Fig. 4, assuming hydraulic diffusivity *D* = 0.04 m^2^/s (based on available industry data; see electronic supplement), the effect of pore pressure change, which would have served to inhibit failure, dominates the normal stress change after ≈25 days in a homogeneous model, assuming hydraulic communication between the production horizon and the fault. The predicted (negative) normal stress change, however, increases sharply at ≈80 days as the pressure front approaches the WWF, approaching 0.1 MPa, potentially promoting strike-slip failure. Although subsurface pore pressure data are not available to confirm this hypothesis, we propose that the minor vertical fault (un-named but imaged by drilling logs as shown in Fig. 2a, and indicated by dot-dashed line in Fig. 2b) just south of the WWF served as an impermeable barrier that offset the permeable Eocene layer by ≈60–90 m (i.e., greater than the thickness of the productive reservoir sandstones; Fig. 2a) and thereby blocked the direct pore pressure effect on the WWF, such that the undrained normal stress response was dominant. Based on past studies of triggered earthquakes, a reduction in normal stress on the order of 0.1 MPa could have been sufficient to potentially trigger an earthquake (Simpson and Negmatullaev 1981; Stein 1999). Our specific model does require that the Kern County earthquake, either the primary WWF rupture or initial rupture on an adjacent secondary fault, nucleated at a relatively shallow depth, close to 3 km. This depth is admissible within the range of hypocentral depths we inferred for the 1952 earthquake of 2–17 km, although the nucleation depth for this event remains poorly constrained (see electronic supplement).

The simple analytical model presented in this study provides a proof-of-concept, demonstrating that production from well K.C.D.L 85-29 could plausibly have perturbed stresses on the WWF system in a manner that promoted failure within 80–90 days of the start of production. While the spatial extent of stress change associated with production was small, initial unstable rupture in proximity to a major fault could have effectively triggered, or grown into, continuing rupture (e.g., McGarr and Simpson 1997). Cascading (triggered) ruptures associated with initial induced events are presumably inhibited in general because induced events tend to occur at very shallow depths, which are typically characterized by velocity strengthening frictional properties (e.g., Shirzaei et al. 2016). The depth of well K.C.L.D. 85-29, however, was close to the 3–5 km upper cutoff of crustal seismicity that Marone and Scholz (1988) suggested marks the transition from a shallow velocity-strengthening regime to a seismogenic velocity-weakening regime.

## Conclusions

Although uncertainties in epicentral location and (especially) hypocentral depth of the 1952 Kern County earthquake are considerable, our review of available industry records demonstrates that there was a temporal and spatial association between deep production in the Wheeler Ridge oil field and the 1952 Kern County earthquake. The earthquake occurred 98 days after the start of production from Eocene strata at a depth of approximately 3 km; while hypocentral depth of the earthquake is unconstrained (Ishida and Kanamori, 1980), the epicenters estimated by past published studies are within 2 to at most 7 km of the well. We have moreover proposed a mechanism based on simple analytical solutions with geologic structure and key reservoir parameters estimated from available industry data, that provides a plausible explanation for a causative relationship between industrial activities and the earthquake. While no such modeling exercise can be conclusive, and other triggering scenarios could be explored (for example involving nucleation on the Wheeler Ridge fault), our results demonstrate that production from deep Eocene strata could have caused sufficient stress change to perturb the nearby WWF significantly within ≈80 days of the start of production if, as we propose, an intervening secondary fault functioned as a hydrologic barrier. A delay on the order of 3 months would not be unprecedented: In a retrospective study of earthquakes in Oklahoma, Hough and Page (2015) concluded that injection-induced earthquakes commonly occurred within 3–6 months of the time that injection wells were permitted (i.e., 2–5 months of when they went into operation). In general, the time delay between subsurface fault injection or withdrawal and potential triggering is expected to be highly variable, depending on myriad factors including the distance of key wells from active faults.

Our proposed model moreover points to an explanation of why significant earthquakes are not commonly induced by production in proximity to major faults: in general, one expects the direct pore pressure effect to dominate, inhibiting failure, so that the effects of poroelastic stress change will dominate -- thereby promoting failure -- only in cases where a fault seal detailed fault zone structure includes an impermeable barrier between the production layer and the actively deforming fault core. The epicentral region of the Kern County mainshock is within ≈5 km of a 2005 cluster that was recently suggested to be an unusually deep injection-induced sequence with a largest event of M_w_4.6 (Goebel et al. 2016). In that case, Goebel et al. (2016) proposed that a newly recognized Tejon fault provides a high-permeability pathway for injected fluids. While we propose that the 1952 mainshock was induced via a different mechanism, it is possible that the WWF might be especially susceptible to induced earthquakes in this region. Although we cannot rule out the possibility that the 1952 earthquake was purely tectonic in origin, the proximity of the rupture zone for this event to the producing reservoir and the short time lapse between onset of deep production and the occurrence of the 1952 earthquake (98 days) supports the hypothesis that this event was triggered by stress changes associated with oil production.

## Data and resources

Monthly “California Oil Fields: Summary of Operations” published by the State Oil and Gas Supervisor are available from (ftp://ftp.consrv.ca.gov/pub/oil/Summary_of_Operations; last accessed 6 June 2016). Drilling records are available from http://www.conservation.ca.gov/dog/Pages/wellfinder.aspx.

Historical weather reports for the town of Bakersfield, California (approximately 40 km north of Wheeler Ridge), are available from https://www.wunderground.com/history/airport/KBFL/2009/5/10/DailyHistory.html (last accessed 11 July 2017). The global earthquake catalog is available from https://earthquake.usgs.gov/earthquakes/search/ (last accessed 12 July 2017).

## Electronic supplementary material

ESM 1 (DOCX 498 KB).
