# Supplementary material for: Was the Mw 7.5 1952 Kern County, California, earthquake induced (or triggered)?
Source: J Seismol. 2017 Oct 2;21(6):1613–21. doi: 10.1007/s10950-017-9685-x (PMC5693966; doi:10.1007/s10950-017-9685-x)
Supplement: Supplementary file 1 — (DOCX 498 KB). [file 10950_2017_9685_MOESM1_ESM.docx]

**Electronic Supplement to,**

**Was the M_w_ 7.5 1952 Kern County, California, Earthquake Induced?**

Susan E. Hough^1^, Victor C. Tsai^2^, Robert Walker^3^, andFred Aminzadeh^3^

^1^U.S. Geological Survey, Pasadena, California

^2^California Institute of Technology, Pasadena, California

^3^University of Southern California, Los Angeles, California

1. Materials and methods

Background information regarding early industry data, constraints on the epicenter of the 1952 Kern County earthquake, and the analytical poroelastic model.

**Materials and Methods**

**Industry Records and Units**

We preserve non-SI units from industry reports, as they are part of the archival record, and provide an indication of the precision to which quantities were reported. Oil production is reported in standard U.S. oil barrels, equivalent to 42 gallons, or 0.159 m^3^.

Oil density is reported using American Petroleum Institute (API) gravity, a measure of relative density compared to water. The initial oil production from well K.C.D.L. 85-29 was 35.3 API (Walling, 1952), corresponding to a density of 850 kg/m^3^. A resistivity log indicates a formation resistivity, *F*, of approximately 200-400 ohm-m for this horizon. This implies a porosity, *φ*, on the order of 6-8% assuming *F=a*/*φ^m^*, assuming nominal values a=1.0 and m=2.0 (Archie, 1942).

Oil industry record-keeping for well locations is based on the Public Land Survey System, developed and first used in the United States in the late 18^th^ century to divide public lands for sale and settlement. The system remains in use in most of the United States, defines townships as parcels of land 36 miles square relative to established baselines and meridians, specified by Township (distance north or south relative to a baseline) and Range (distance east or west.) Townships are divided into 36 sections, each approximately one square mile. A specified Township, Range, and Section therefore provides a given well location to the nearest 1.6 km. Precise well locations in California are generally available from the California State Division of Oil, Gas, and Geothermal Resources. Individual wells have API (American Petroleum Institute) numbers that provide a unique and permanent numeric identifier for every well drilled for oil and gas in the United States. Precise well locations for individual wells with known API numbers are available from the California Division of Oil, Gas, and Geothermal Resources. Precise wellhead locations, surveyed to an accuracy of < 1 m, were included in original drilling records for every well. The surface locations of the wells shown in Figure 2a are thus known precisely; wells are assumed to be vertical at depth as deviation logs for these wells were not available. Locations of lithologic units and fault strands in the upper ≈3 km are constrained by well logs; the location of White Wolf fault (WWF) below this depth is inferred.

**Kern County Epicenter**

The epicenter of the 1952 Kern County mainshock has been estimated by several different studies starting with Gutenberg (1955), who determined an epicenter location of 35.00N, -119.033W (Figure S1). Revisiting available data for the mainshock and its aftershocks with more modern methodology, Ishida and Kanamori (1980) estimated an epicentral location of 34.977N, 119.033W. The modern catalog magnitude and location are Mw 7.5, 34.9582N, 118.998W (Felzer, 2013), as estimated by Hutton *et al*. (2010), and subsequently used in the Uniform California Earthquake Rupture Forecast version 3 (UCERF3). Although the UCERF3 catalog is generally regarded as the definitive catalog for California, the catalog location for the Kern County mainshock was estimated via a systematic relocation of the entire Southern California Seismic Network catalog (Hutton *et al*., 2010). An epicenter estimated using a systematic relocation approach applied to the entire catalog is not expected to be as reliable as the results from focused investigation that considers carefully the unique data set available for a large earthquake (Kate Hutton, personal communication, 2016). While the different solutions to some extent reflect inherent uncertainty associated with the limited early data, the location estimated by Ishida and Kanamori (1980) is thus considered preferred over the current catalog location, with a horizontal uncertainty on the order of 3-5 km. In all of these studies, the depth of the 1952 event is found to be effectively unconstrained.

To investigate the hypocentral location further, we have reanalyzed the available phase data included in Gutenberg (1955), first confirming the Ishida and Kanamori (1980) conclusion that the mainshock phase data do not provide a reliable depth estimate. We also reanalyzed phase data presented by Richter (1955) for an aftershock on 25 May 1953, the location of which Richter identifies as indistinguishable from the mainshock. Phase picks from a total of 11 stations are available for this aftershock, including three portable stations installed following the mainshock. Analyzing these data using the one-dimensional velocity model and methodology now used by the network (Hutton *et al*., 2010), we estimate an epicenter of 35.00N, 119.02W. The optimal depth is 11.4 km (with an average rms misfit to travel time picks of 0.11 s); average rms misfit is < 0.15 s for a depth range of 2-17 km when uncertainties in the velocity model are accounted for. Given the inaccuracy of early clocks, data are insufficient to constrain depth within the range 2-17 km and even this large range is unlikely to capture full uncertainties. As illustrated by the inset panel in Figure S1, the UCERF-3 catalog epicentral location is several km away from this location, while the preferred estimates from earlier studies are significantly closer.


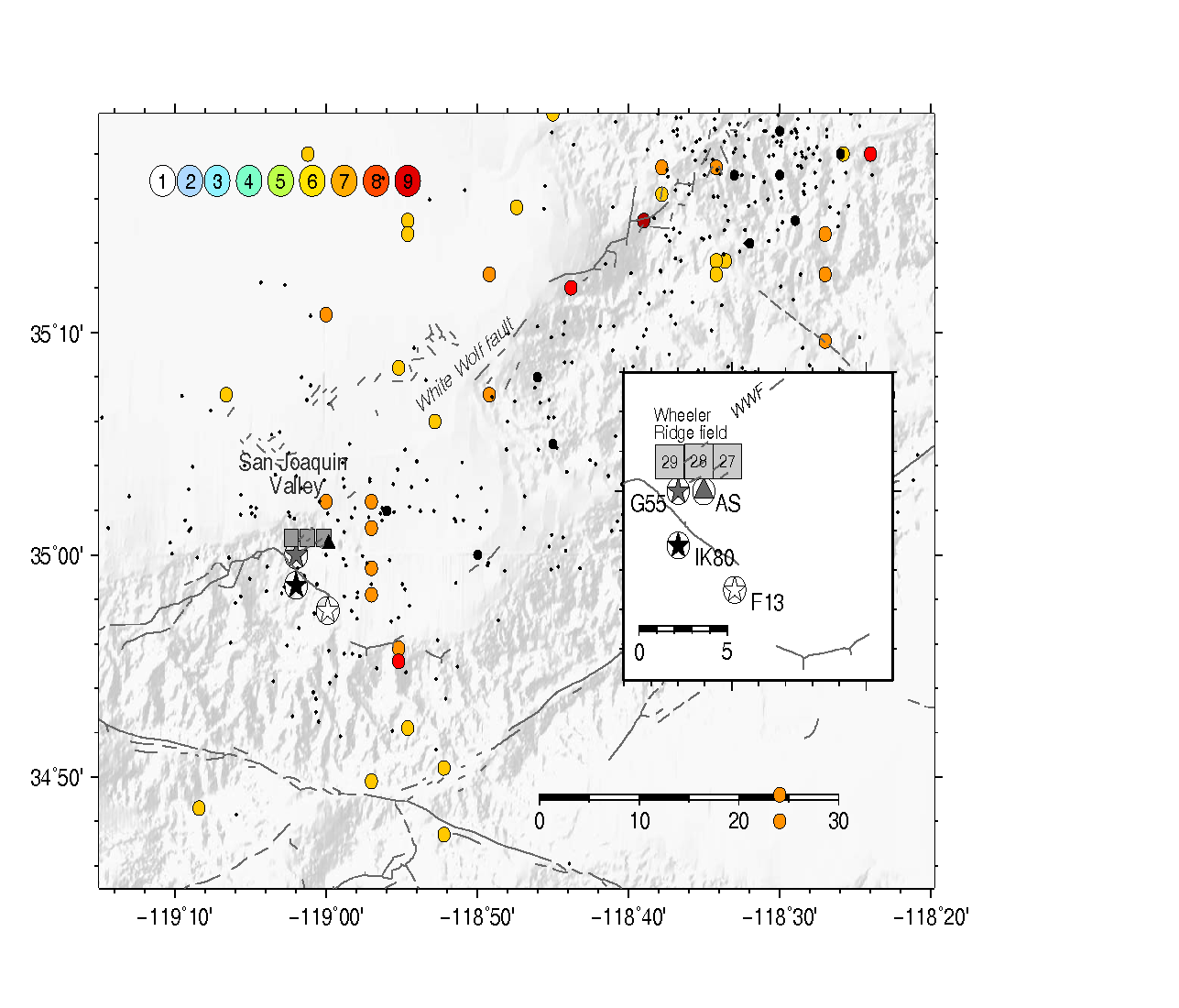


**Figure S1.** Map showing locations of Wheeler Ridge oil field operations (small gray squares) in the early 1950s, locations of aftershocks following the 1952 mainshock from the SCSN catalog (small dots) and Dreger and Savage(1999) (larger black dots), estimated modified Mercalli intensities for the mainshock from the NOAA database (see Data and Resources; colored circles), and location of a 1939 M_w_4.6 event (4) (triangle). Inset panel shows Sections in Township 11N, Range 20W within the Wheeler Ridge Oil Field. Both figures include published Kern County mainshock epicenters from Gutenberg (1955), Ishida and Kanamori (1980), and Felzer (2013) (respectively circled gray, black, and white stars labeled G55, IK80, and F13). The inset includes our relocated epicenter of the 25 May 1953 aftershock (circled triangle labeled AS).

Lastly, geological investigations following the mainshock provide independent evidence for the location and depth extent of rupture in the vicinity of Wheeler Ridge. Buwalda and St. Amand (1955) describe the geological effects of the earthquake, including landslides as well as inferred tectonic surface rupture, and include a detailed discussion of geological effects near the western end of Wheeler Ridge. They describe three types of ruptures on Wheeler Ridge: 1) soil cracks described as “often tens of feet long”; 2) fractures interpreted as the result of landsliding; and 3) “long straight [breaks] which crossed the crest of the hills obliquely on the projection and trend of the fault. Unlike the two previous types, these breaks were independent of topography, traversing hills and depressions indifferently.” The locations of these breaks, which trend N50-55E and extend for a total of ≈2 km, are described in detail and shown on the accompanying map (Figure S2). Their alignment is within ≈5° of mapped secondary fault traces (Buwalda and St. Amand, 1955). The dominant movement on the breaks was vertical, with as much as 1.3 m (northwest side down) displacement. The southwestern terminus of the breaks is less than 2 km northeast of the epicenter estimated by Gutenberg (1955), and less than 2 km southwest of well 85-29. Buwalda and St. Amand (1955) concluded that the breaks were “the surface expression of fractures and sharp distortion which have extended steeply upward through the Wheeler Ridge overthrust plate from the trace of the White Wolf fault below it.” While this statement is perhaps ambiguous, the description of the breaks is consistent with tectonic surface rupture on either the WWF or a secondary fault in proximity to the main fault.


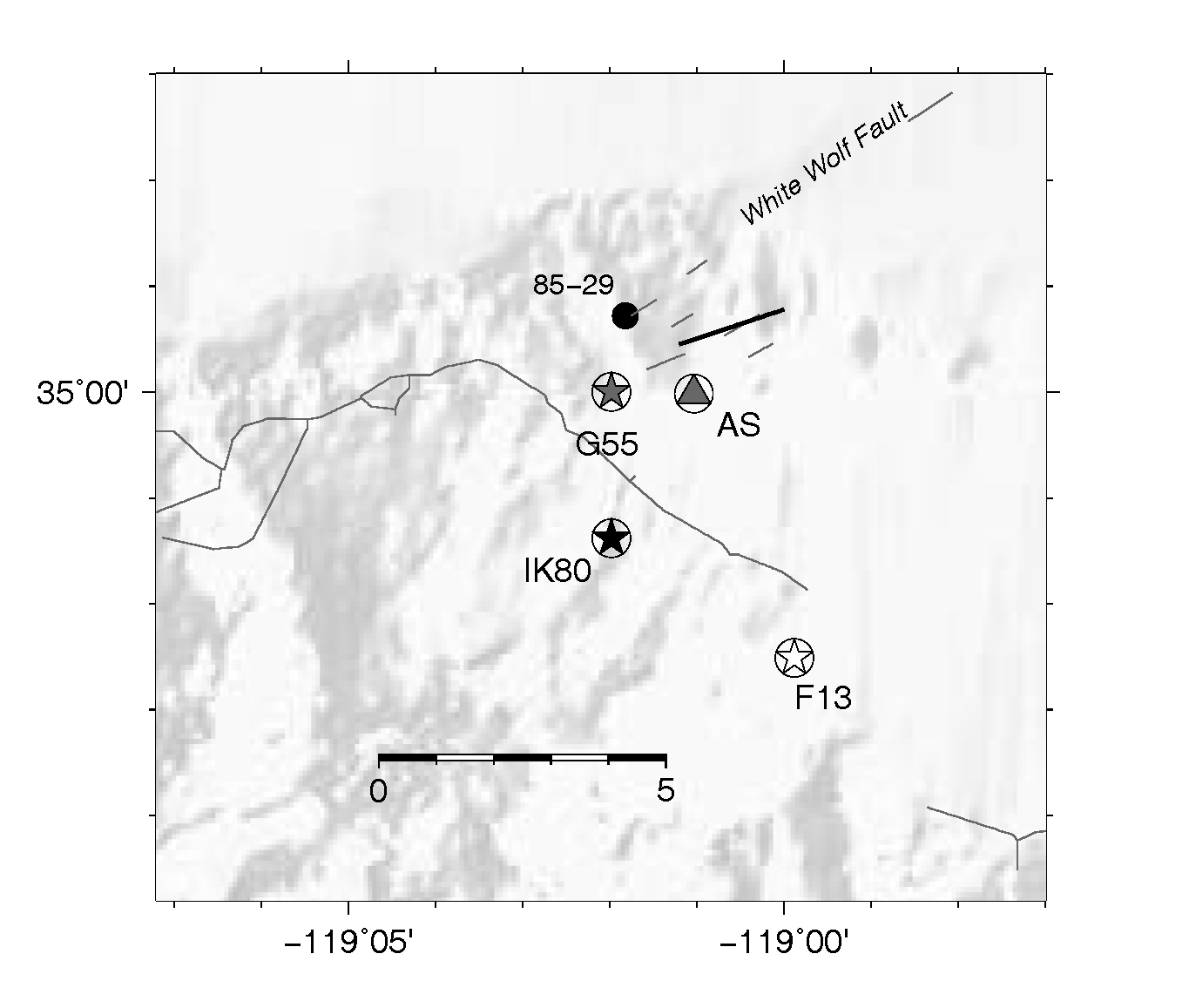


Figure S2. Wheeler Ridge region including published Kern County mainshock epicenters from Gutenberg (1955), Ishida and Kanamori (1980), and Felzer (2014) (respectively circled gray, black, and white stars labeled G55, IK80, and F13); our relocated epicenter of the 25 May 1953 aftershock (circled triangle labeled AS); location of well 85-29 (black dot), mapped faults (Jennings, 1994), and surface breaks mapped by Buwalda and St. Amand (1955) (heavy black line).

**Analytical Solution**

Changes in normal stress as well as changes in pore pressure on a fault oriented approximately perpendicular to a reservoir from which fluid is extracted can be estimated with analytical solutions. Although these analytic results are not expected to be accurate due to over-simplification, including neglect of 3-dimensional effects in fault/reservoir geometry and in-situ stress field, they provide an order-of-magnitude understanding of how large the two competing effects would have been on the WWF. A simplified model can thus be used to test the hypothesis that oil production could have brought the WWF significantly closer to failure during the time between the beginning of production on 14 April 1952 and the time of the Kern County earthquake on 21 July 1952.

To estimate the changes in normal stress acting on the fault, we assume that the loading can be approximated by a single line source of pressure *Δp(t)* acting on a vertical thickness *H*. Furthermore, it is assumed that the medium is a homogeneous half space to the right of the loading (see Fig S3). While this is an approximation to the actual loading situation, analytical and semi-analytical models can be used to estimate the actual stress changes provided that the location of interest is sufficiently far from the actual free surface as well as the model free surface (e.g., Soltanzadeh and Hawkes, 2008). For the WWF scenario, loading on the nearest part of the fault satisfies both of these assumptions.

Figure S3. Schematic of model. Variables are described in text.

For a single line source with force per unit distance *F* acting in the *x* direction, and in the absence of an ambient (tectonic) stress, the normal stress *σ_xx_* due to reservoir pressure is given by

* σ_xx_*=2*F*/(π*L*)*cos^4^(*θ* 

where *L* is distance from the force and *θ* is the angle from perpendicular. Integrating over vertical thickness *H* yields

*σ_xx_*=2*Δp*/(π*L*)*[2*L***H*/(4*L*^2^+*H*^2^) + cot^-1^(2*L*/*H*)] (A2)

where *Δp* is pressure and σ_xx_ is evaluated at x=L, y=0, along the midpoint of the pressure source. This expression thus provides an estimate of the magnitude of the normal stress change due to a given pressure source of height H.

To estimate the appropriate value of *L* and *Δp*, we use a characteristic length scale and timescale for diffusion of pore pressure from a source of constant production rate Q,

 *Δp*=*Q*/(4p*DHc_t_* (A3)

where *D* is diffusivity and *c_t_* is matrix compressibility, and *L(t)*=*L_0_*-2sqrt[*Dt*]

where *L_0_* is initial horizontal distance from the production point to the point of interest (i.e. the fault; i.e., *L_0_*≈1 km), and t= time since production began. Using these expressions in Eq A1 yields an analytic estimate of the normal stress change on a vertical plane as a function of time since production. We note that the sign of the normal stress change due to a reduction in pore pressure (*Δp*) is to reduce the normal stress and therefore bring a fault with this orientation towards failure. For this estimate, we ignore the dip of the WWF, taking its value to be close to 90 degrees.

To estimate the direct pore pressure effect, which for production tends to increase the effective normal stress and hence bring the fault farther from failure, we use the analytic solution for pore pressure change in a homogeneous halfspace

p’=C*erfc[L_0_/(2sqrt(Dt))] (A4)

To approximately account for the boundary condition being one of constant flux, we estimate C such that the pressure perturbation at distance 2sqrt[Dt] is equal to the characteristic pressure given by Eq A2. With this estimate, then

p’=*Δ*p*erfc[L_0_/2sqrt(Dt)]]/erfc(1) (A5)

where *Δ*p is as above.

**Estimation of Parameters**

The modeling results presented in this study depend critically on the assumed value of diffusivity, which in turn depends on assumed values of permeability, *k*, dynamic viscosity, , and matrix compressibility, *c_t_*: *D* = *k*/(*μc_t_*). Several of these parameters are potentially uncertain by orders of magnitude, and are estimated by considering available well log data and information. We estimate a dynamic viscosity of 2*10^-3^ Pa-s (Glaso, 1980) (corresponding to a kinematic viscosity of 3*10^-6^ m^2^/s assuming a density of 850 kg/m3). We estimate permeability towards the high end of the typical range for sandstones (e.g., Ahmed, 2006), 10^-13^ m^2^ based on well log indication of “good permeability and porosity” within the Eocene production horizon. Fitts (2013) estimates average compressibility of 5*10^-9^ Pa^-1^ based on reported values for sandstones. Based on the Calhoun correlation assuming isothermal conditions (Calhoun, 1975) we estimate 6.2*10^-10^ Pa^-1^ for the reservoir sandstone. Assuming the other parameters and this range in compressibility, we estimate *D* = 0.01 – 0.08 m^2^/s, and take 0.04 m^2^/s as the preferred value for our calculations. Layer thickness, *H*, is constrained from the drill log to be 47 m. Assuming *D* = 0.01 m^2^/s, the predicted normal stress change on the WWF would be larger, but would increase sharply after ≈300 days rather than 80 days. If diffusivity is higher than 0.04 m^2^/s, the predicted normal stress change on the WWF would have acted on the fault for a longer period of time. Our precise model results are uncertain, and more complicated models could be explored, including triggering of an initial nucleation on other fault strands in proximity to the wells drilled in 1951-1952. Our proposed mechanism assuming a simple model and best estimates of parameters can, however, plausibly explain the occurrence of the 1952 earthquake 98 days after the start of production. Fundamentally, it provides a proof of concept that stresses could have been perturbed significantly to promote failure on the nearby WWF within months following the onset of production.

**References**

Ahmed, T.H. (2006). Reservoir engineering handbook, Gulf Professional Publishing, 1376 pp.

Archie, G.E. (1942). The electrical resistivity log as an aid in determining some reservoir characteristics, *Trans. AIME* **146:1**, 54-62.

Buwalda, J.P. and P. St. Amand (1955). Geological effects of the Arvin-Tehachapi earthquake, in Oakeshott, G.B., ed., Earthquakes in Kern County California during 1952, *California Div. Mines. Bull.*, **171,** 41-56.

Calhoun, J.R. (1976). Fundamentals of reservoir engineering, University of Oklahoma Press, Norman.

Dreger, D. and B. Savage (1999). Aftershocks of the 1952 Kern County, California, earthquake sequence, *Bull. Seism. Soc. Am.* **89:4**, 1094-1108.

Felzer, K.R. (2013). The UCERF3 earthquake catalog, Appendix K in The Uniform California Earthquake Rupture Forecast, Version 3 (UCERF3), The Time-Independent Model, *U.S. Geol. Surv. Open-File Rep.* 2013-1165.

Fitts, C.F. (2013). Groundwater science, Academic Press, 696 PP.

Glaso, O. (1980). Generalized pressure-volume-temperature correlations, *J. Petroleum Techn.* **32:5**, 785-795.

Gutenberg, B.G. (1955). Epicenter and origin time of the main shock on July 21 and travel time of major phases, in Oakeshott, G.B., ed, Earthquakes in Kern County, California, during 1952, *California Div. Mines. Bull*., 171, 157-164.

Hutton, K., J. Woessner, and E. Hauksson (2010). Earthquake monitoring in southern California for seventy-seven years (1932-2008), *Bull. Seism. Soc. Am*., **100:2**, 423-446, doi:10.1785/0120090130.

Hutton, K., oral communication, 2016.

Ishida, M. and H. Kanamori (1980). Temporal variation of seismicity and spectrum of small earthquakes preceding the 1952 Kern County, California, earthquake, *Bull. Seism. Soc. Am*., **70:2,** 509-527.

Jennings, C.W. (1994). Fault activity map of California and adjacent areas, with locations and ages of recent volcanic eruptions, California Div. Mines and Geology.

Richter, C.F. (1955). Foreshocks and aftershocks, in Oakeshott, G.B., ed., Earthquakes in Kern County California during 1952, *California Div. Mines. Bull.*, **171**, 177-197.

Soltanzadeh, H. and C.D. Hawkes (2008). Semi-analytical models for stress change and fault reactivation induced by reservoir production and injection, *J. Petrol. Sci. and Eng*., **60:2**, 71-85.

Walling, R.W. (1952). Operations in District No. 4, 1952, in California Oil Fields, Summary of Operations, pp 72-84, V. 38:2.
